# The current status and effects of emergency drug shortages in China: Perceptions of emergency department physicians

**DOI:** 10.1371/journal.pone.0205238

**Published:** 2018-10-09

**Authors:** Caijun Yang, Wenfang Cai, Zongjie Li, Amy Theresa Page, Yu Fang

**Affiliations:** 1 The Department of Pharmacy Administration and Clinical Pharmacy, School of Pharmacy, Xi'an Jiaotong University, Xi’an, China; 2 The Center for Drug Safety and Policy Research, Xi'an Jiaotong University, Xi’an, China; 3 Western Australian Centre for Health and Ageing, University of Western Australia, Perth, Australia; 4 Centre for Medicine Use and Safety, Monash University, Perth, Australia; La Trobe University, AUSTRALIA

## Abstract

**Objectives:**

The shortage of emergency drugs in China is severe. This study aimed to characterize emergency drug shortages in China and to measure their effects.

**Methods:**

An online questionnaire based on a literature review was sent to emergency department physicians in Chinese secondary and tertiary hospitals from November 2016 to February 2017. The survey asked physicians questions about their experiences with emergency drug shortages.

**Results:**

In total, 236 physicians from 29 provinces participated in the survey. According to their responses, 90.7% of the respondents experienced drug shortages during the last year. More than half of the physicians (65.7%) reported that drug shortages occurred at least once a month. Hospitals in the eastern and western regions of China had more emergency drugs in shortage than hospitals in central China, especially those with many inpatient beds (≥800). In addition, the shortage situation was more serious in secondary hospitals than in tertiary hospitals. More respondents agreed that original medicines, injections, essential medicines, medicines without alternative agents and cheap medicines were more susceptible to shortages than generics, oral medicines, nonessential medicines, medicines with alternative agents and expensive medicines, respectively. Most respondents thought that drug shortages always, often or sometimes affected patients [delayed therapy (62.6%), longer rescue and recovery times (58.9%) and higher costs (58.7%)] and physicians [inconvenience (81.0%), higher pressure (76.5%) and harm to patient-doctor relationships (72%)] and compromised hospital reputations (55.1%).

**Conclusions:**

The shortage of emergency drugs in China is serious, especially in secondary hospitals located in eastern and western China. Emergency drug shortages have significant effects on patients and physicians.

## Background

A drug shortage refers to a situation in which a drug cannot meet the current or projected demand at the user level [1]. Over the last few decades, drug shortages have become a complex global health problem affecting both developed and developing countries [2]. Developed countries in Europe and North America have reported drug shortages, and 154 new shortages were reported in 2016, even after recent efforts by the U.S. government to solve this problem [3]. European pharmacists across 36 countries experienced drug shortage problems in 2014, and most reported that this problem occurred on a daily or weekly basis [4]. Ireland reported shortages of 64 drugs during a four-month period with an average duration of more than two weeks [5]. Developing countries have been heavily affected by drug shortages and many of the affected drugs are essential medicines. In India, 130 medicines on the Essential Medicines List and Rationalized Drug List were reported to be in shortage in 2013 [6]. In China, researchers identified 148 drugs in shortage from 2006 to 2015, most of which were cheap and essential medicines [7].

Drug shortages affect all stakeholders in the healthcare system, especially patients and healthcare workers. Drug shortages can lead to suboptimal patient care, delayed or inadequate treatment, or cancellation of surgery [8, 9]. Because of these shortages, patients may experience medication errors, adverse drug reactions, a higher risk of drug toxicity, longer recovery times, or increased healthcare costs due to the use of a less effective substitute drug [10–12]. Among healthcare workers, pharmacists and physicians are the directly affected groups. Pharmacists are the first professionals who face drug shortages in hospitals. The time required to negotiate with manufacturers and wholesalers detracts from clinical and quality improvement tasks [13]. In collaboration with pharmacists, physicians make choices to ration the limited remaining supply and choose alternative medicines [14]. Consequently, drug shortages can undermine the clinical relationship between physicians and patients. Moreover, the reputation of the health service is negatively affected when the service cannot provide necessary drugs for its patients.

Shortages of drugs used in emergency departments and in the prehospital setting (referred to as emergency drugs [15]) have more severe consequences. The unavailability of emergency drugs can result in poor health outcomes because alternative drugs may be less effective or have poorer safety profiles than the preferred medicine [16]. Because healthcare workers may be unfamiliar with using these agents, more medication errors may occur [17]. Treatments are delayed when no alternative drugs are available, which can cause poor health outcomes and patients being denied treatment. Moreover, the relationship between physicians and patients is adversely affected more frequently.

The shortage situation for emergency drugs is severe, especially in China. In 2015, many hospitals reported shortages of pralidoxime chloride and noradrenaline [18]. In 2011, protamine was in short supply, which seriously affected the ability to perform certain surgeries, such as extracorporeal circulation surgeries for patients with heart disease [19]. One study that investigated drug shortages in the hospitals of six Chinese provinces concluded that shortages were higher for emergency drugs than for other types of medicines [20]. Emergency drug shortages may occur because emergency drugs are more susceptible to shortages than other drug types, as most emergency drugs have the characteristics of shorter expiry dates and lower and unstable demands. In addition, the whole-market requirements are very low, even though every hospital has to prepare a certain number of emergency drugs in advance. The low prices of most emergency medicines in China have worsened the situation because the production companies and wholesalers do not maintain sufficient stocks [21].

The Chinese government has tried to address this emergency drug shortage problem. The Chinese National Health and Family Planning Commission announced a policy in October 2011 to improve the supply of emergency drugs and medicines for infectious diseases [22]. This policy required local governments to address the drug shortage problem through a multifaceted intervention that included gathering data on shortages, building an information platform and improving hospital inventory management. Efforts were redoubled in 2015 with the implementation of another policy to address the emergency drug supply [23]. In China, each province has a drug tender bidding center to select drug manufacturers and to determine drug prices for government-run healthcare institutions using a ‘two-envelope’ system. The first envelope is quality, and the second envelope is price (the lowest price wins). A composite score is calculated to determine the winner. In some provinces, one company wins the tender, whereas in other provinces up to three companies can win. To win the tender, the manufacturers usually offer a very low price that will not change for a long time (typically 5 years for most provinces). Lower prices and fewer manufacturers are regarded as one important reason for drug shortages. Therefore, this 2015 policy allowed all public hospitals to order emergency drugs from manufacturers who met the technical and quality requirements, although the hospitals themselves needed to negotiate the price. However, the shortage of emergency drugs has not improved. In May 2017, Guangdong province faced a shortage of 18 emergency drugs when suppliers did not respond to state tenders, including tetanus antitoxin and naloxone hydrochloride [24]. Other provinces experienced the same problem. Therefore, the shortage of emergency drugs is a public health issue in China.

The ongoing emergency drug shortage crisis has exacted a significant effect on patient safety. Clarifying what specific types of emergency drugs have been in critical shortage and demonstrating the effects of emergency drug shortages is pivotal for coping with or finding solutions for this problem. However, to the best of our knowledge, research is limited on the exact causes of and solutions for drug shortages in China [21]. Limited research has reported the emergency drug shortage situation or its effects on patients and health professionals. This study aimed to analyze and characterize emergency drug shortages and explore their impacts in China.

## Methods

### Participants

The study participants were physicians working in hospital emergency departments. This group was selected mainly because emergency physicians were directly affected by drug shortages, worked in emergency settings and used emergency drugs frequently. Therefore, they were familiar with the effects of drug shortages on hospitals, workers and patients.

### Survey design

We used a questionnaire survey to obtain data on the perceptions of physicians who were faced with emergency drug shortages in China. A literature review was performed to develop the survey questionnaire [25], which was refined by two experts in the field (S1 Text). Then, we edited and published the questionnaire in SO JUMP, which is a professional platform in China for online questionnaires. The survey was piloted by five emergency physicians and then refined based on feedback.

The final survey included eight questions addressing the following topics: the details of the hospital where the respondent worked (including the hospital location, hospital level and type of hospital); the status of emergency drug shortages (including the frequency of these shortages and the names of the shortage drugs); the characteristics of these shortage drugs (whether particular drug classes were more affected by shortages than others); and the effect of shortages on hospitals, healthcare workers and patients.

To determine the status of emergency drug shortages, we asked how often drug shortages occurred and the names of the drugs in shortage. We listed 25 types of emergency drugs from which the respondents could choose. These 25 drugs were selected using the following sources and steps. Source 1 was a drug list containing 43 emergency drugs published by the Chinese National Health and Family Planning Commission in 2015 to encourage all public hospitals to order these drugs directly from manufacturers to solve the shortage problem [26]. Source 2 were four additional drug shortage lists published by four different provinces (Beijing, Tianjin, Jiangsu, and Gansu) in 2016, each of which contained different types of drugs in shortage (including emergency drugs) in that specific province. A drug was selected for the survey if it appeared on all five lists, resulting in a final list of 25 drugs.

To determine the characteristics of the drugs in shortage, we asked whether particular drug classes were more affected by shortages than others, including original medicines or generics, orals or injections, essential medicines or nonessential medicines, medicines with or without alternative agents, and cheap medicines or expensive medicines. Notably, among the 25 drugs selected, most were essential, cheap medicines without alternatives. More specifically, 22 drugs were on the National Essential Medicine list. All of these drugs had a daily cost of less than 15 RMB (1RB = $0.1594), and 10 of them had costs of no more than 5 RMB. Except for two medicines (sodium dimercaptopropane sulfonate and acetamide), no complete substitute existed for these drugs.

To determine the effects of drug shortages on hospitals, healthcare workers and patients, a five-point Likert scale was used to measure the perceptions of emergency physicians. This section contained 17 subquestions.

### Recruitment and screening

Participants were recruited using multiple strategies. First, valid email addresses of 968 emergency physicians who published papers from 2008 to 2016 were found in the China National Knowledge Infrastructure database. Email invitations were sent to these emergency physicians with a link to request their participation in the survey. Second, a social media platform for national emergency workers was identified, and an invitation to participate was sent to emergency physicians via this group. Third, we asked the respondents to distribute our survey to other emergency physicians in all of the invitations. The survey was launched on November 15, 2016, and was concluded on February 15, 2017. To encourage survey completion, three reminders were sent to the participants before the closing date.

To ensure eligibility of the respondents, before the formal survey, we displayed an explanatory statement and asked two questions to prevent other groups of people from filling out the questionnaire. In the explanatory statement, we explained the objective of the study. Once the respondents understood the study and agreed to participate, two questions were presented. The first question was “how long have you worked in the emergency department” and the second question was “what is your position (physician, pharmacist, nurse, or other)”. Only physicians who worked in the emergency department for at least 1 year could begin to fill in the questionnaire (as one question pertained to collecting the trade names of drugs in shortage during the last year).

### Statistical analysis

Data were compiled in Microsoft Excel and analyzed by two authors using SPSS 19.0. The descriptive statistical analysis results are reported as ratios and means. The status of emergency drug shortages was indicated by two measures: the frequency of drug shortages and the number of drugs in shortage. For the frequency of drug shortages, we calculated the number of respondents who chose different answers and reported the ratio. For the number of drugs in shortage, we calculated the number of drugs provided by each respondent and reported the mean.

The status of emergency drug shortages was then compared between different geographical regions (i.e., eastern, central and western China) based on their classifications in the China Health Statistical Yearbook. First, a simple comparison was conducted. A Chi-square test was applied to determine the influence of geographical location or hospital level on the frequency of emergency drug shortages. The significance level was 0.05. Because the number of shortage drugs in each hospital was not normally distributed, the Kruskal-Wallis H test was adopted to check whether the number was correlated with the geographical location or the hospital level. The significance level was adjusted to 0.0167, and the Mann-Whitney U test was adopted for this comparison. Second, multivariate analysis was used to examine regional differences by controlling for the hospital type, the number of inpatient beds and the hospital level. We were able to conduct subgroup comparisons based on the results.

As some of the respondents may have been from the same hospital, we also performed a sensitivity test to exclude the cluster effect. If several participants shared the same hospital information, we randomly chose one participant’s data for analysis in the sensitivity test.

### Ethics

Ethical approval was obtained from the Ethics Committee of Xi’an Jiaotong University Health Science Center (Xi’an, China) (Number: 2015–269). All participants agreed to participate in the study, and the data were analyzed anonymously.

## Results

### Demographic characteristics

A total of 236 emergency physicians participated in the study. In total, physicians from 29 of 31 provinces (municipalities and autonomous regions) in mainland China (exceptions were Tibet and Qinghai) were included. For the comparisons, all of these provinces or municipalities were classified into three regions representing eastern (Beijing, Tianjin, Hebei, Liaoning, Shandong, Shanghai, Jiangsu, Zhejiang, Fujian, Guangdong, and Hainan), central (Shanxi, Jilin, Heilongjiang, Anhui, Jiangxi, Henan, Hubei, and Hunan) and western (Sichuan, Guizhou, Yunnan, Tibet, Shaanxi, Gansu, Ningxia, Xinjiang, Guangxi, and Inner Mongolia) China.

As shown in Table 1, 30.9% of the respondents worked in eastern China, 28.4% worked in central China, and 40.7% worked in western China. The emergency physicians were primarily from tertiary hospitals (184/236, 78.0%). The hospital types included general hospitals, specialized hospitals, traditional Chinese medicine hospitals, minority hospitals and traditional Chinese medicine and Western medicine (TCM-WM) hospitals. Most hospitals had more than 800 inpatient beds (154/236, 65.2%).

**Table 1:** Characteristics of the hospitals where the respondents worked.

Characteristics	Respondents (n = 236)
Hospital location (n, %)	
Eastern China	73 (30.9)
Central China	67 (28.4)
Western China	96 (40.7)
Hospital level (n, %)	
Secondary hospitals	52 (22.0)
Tertiary hospitals	184 (78.0)
Hospital type (n, %)	
General	203 (86.0)
Specialized	9 (3.8)
Traditional Chinese Medicine	18 (7.6)
Minority	1 (0.4)
TCM-WM	5 (2.2)
Number of inpatient beds (n, %)	
<100	3 (1.3)
100–199	11 (4.7)
200–499	21 (8.9)
500–799	47 (19.9)
≥800	154 (65.2)

### The status of drug shortages

(1) The frequency of drug shortages

**Fig 1:**
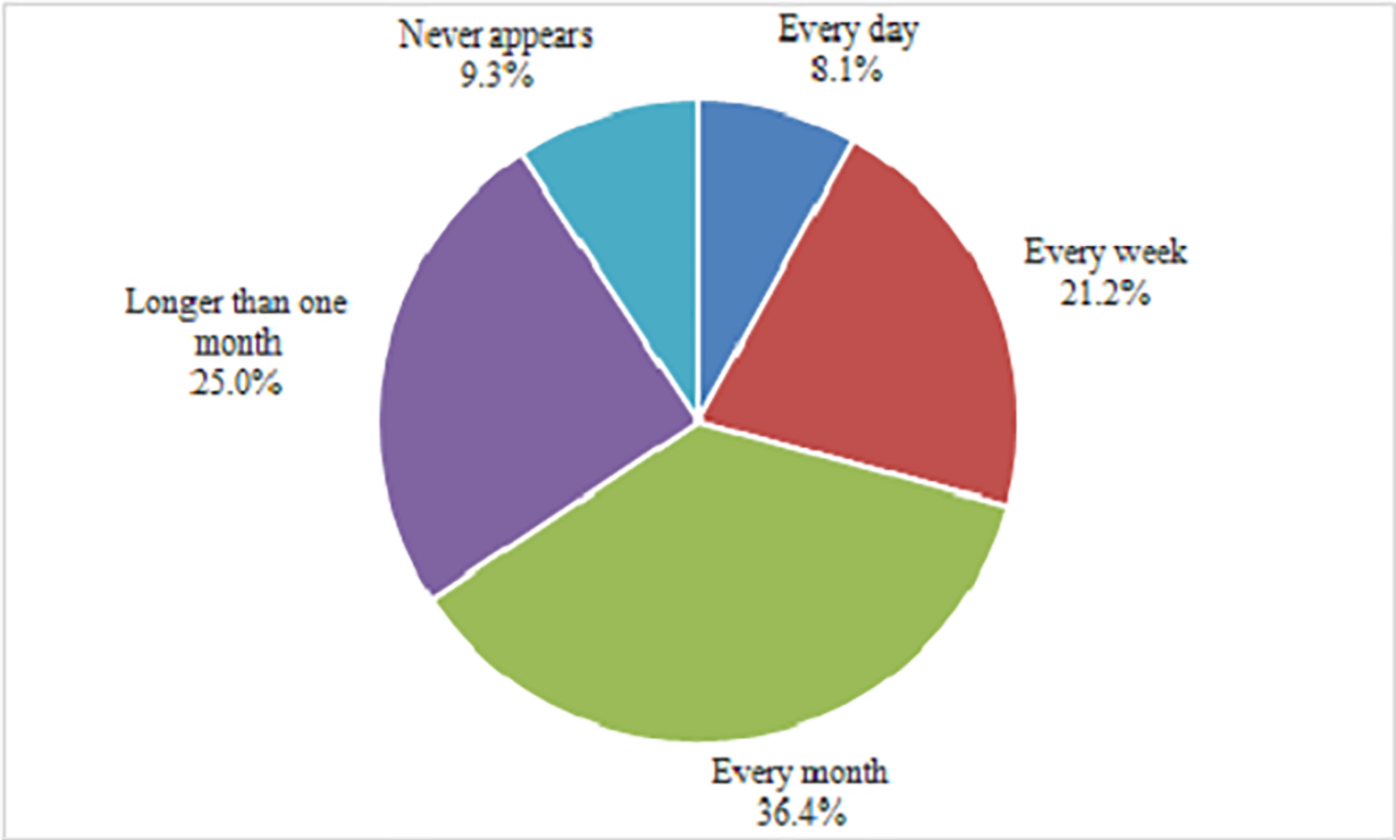
The frequency of drug shortages.

Among the respondents, 90.7% of the emergency physicians had experienced drug shortages during the last year (Fig 1). In response to the question “how often does a drug shortage occur in your department on average”, nearly two-thirds of the physicians (65.7%) reported that drug shortages occurred every month or more frequently. No significant differences in the frequencies of drug shortages existed among the different regions (χ^2^ = 5.920, P = 0.656) or hospital levels (χ^**2**^ = 8.172, P = 0.085) according to the Chi-square test.

In the multivariate analysis, among the four independent variables (hospital type, number of inpatient beds, hospital level and geographical location), only the number of inpatient beds was a significant factor (P = 0.003) affecting the frequency of drug shortages. Then, we divided all of the hospitals into two subgroups for subsequent analysis. Hospitals with less than 800 inpatient beds were included in group 1, and the other hospitals were included in group 2. No significant difference in the frequency of drug shortages was found in the group 1 (χ^2^ = 10.080, P = 0.240) or group 2 (χ^2^ = 5.688, P = 0.692) hospitals among the different regions.

(2) The number of drugs in shortage

**Fig 2:**
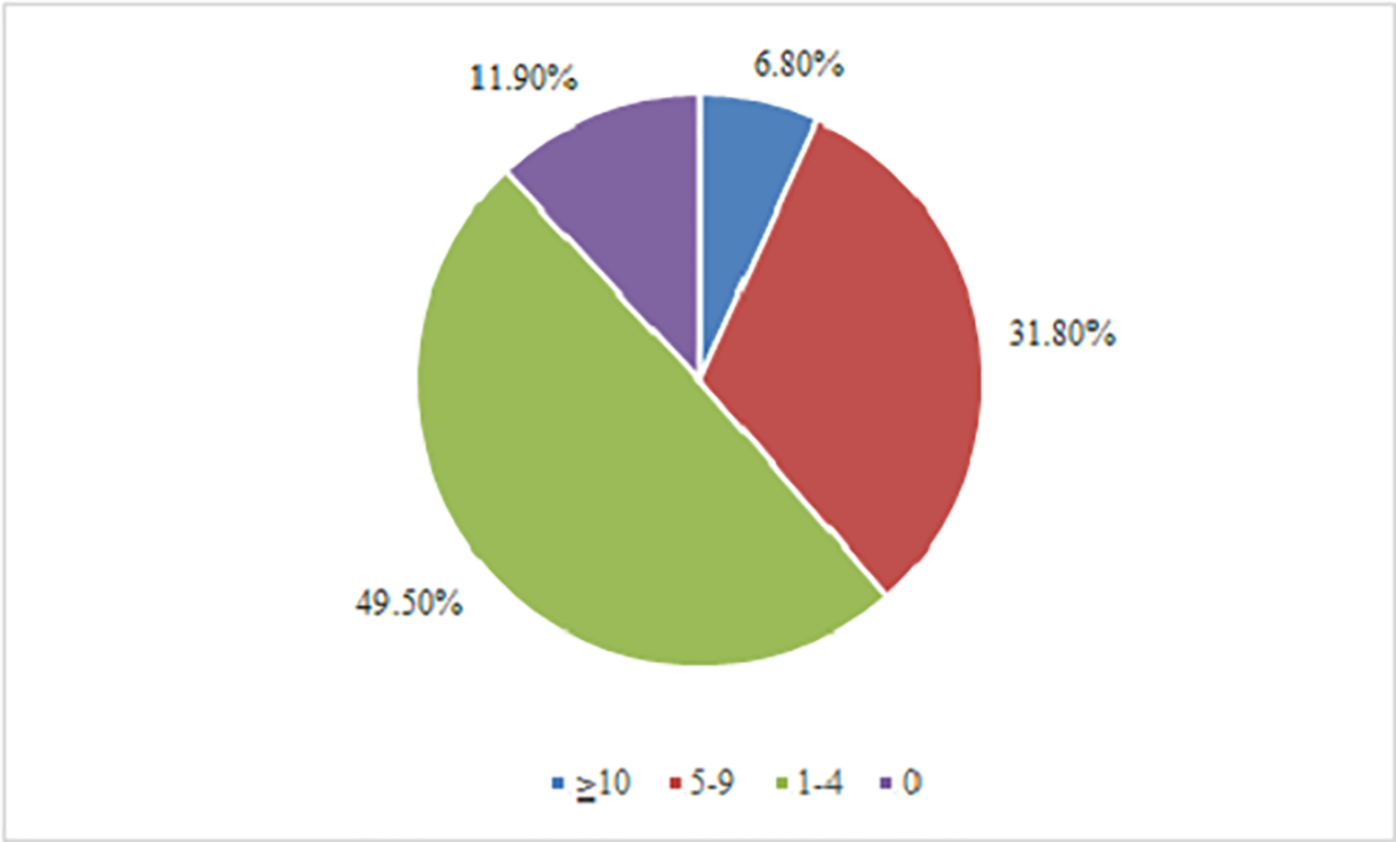
The number of shortage drugs reported by the respondents.

All of the 25 listed emergency drugs in the questionnaire were reported to be in shortage during the last year. As shown in Fig 2, 38.6% of the emergency physicians reported at least five emergency drugs in shortage. The median number of emergency drugs in shortage was four, with a maximum of 15 and an interquartile range of 2–6. Furthermore, we compared the numbers of shortage drugs in different regions. The median number of shortage drugs was three in the central region and four in the eastern and western regions. According to the Kruskal-Wallis H test, there was a significant difference in the number of shortage drugs between different regions (χ^2^ = 13.307, P = 0.001). According to the Bonferroni statistical test and the Mann-Whitney U test, a significant difference in the number of shortage drugs occurred between the eastern and central regions (U = 1636.000, P = 0.001) and between the central and western regions (U = 2333.00, P = 0.003), but no significant difference was found between the eastern and western regions (U = 3336.500, P = 0.593). Similarly, the median numbers of shortage drugs were five and three in the secondary and tertiary hospitals, respectively, which represented a significant difference (U = 3610.00, P = 0.007).

In the multivariate analysis, among the four independent variables (hospital type, number of inpatient beds, hospital level and geographical location), only the number of inpatient beds was a significant factor (P = 0.003) affecting the number of drugs in shortage. Similarly, a subgroup analysis was conducted. No significant difference in the number of drugs in shortage existed among the different regions for group 1 (χ^2^ = 2.290, P = 0.318), but a significant difference was observed for group 2 (χ^2^ = 23.402, P = 0.001). Bonferroni and Mann-Whitney U tests showed significant differences in the number of shortage drugs in group 2 between the eastern and central regions (U = 474.000, P = 0.000) and between the central and western regions (U = 904.500, P = 0.000), but no significant difference was observed between the eastern and western regions (U = 1120.000, P = 0.615).

Among the 25 listed medicines, protamine, sodium dimercaptopropane sulfonate, and pralidoxime chloride were the most commonly reported emergency drugs in shortage (Table 2). More than half of the respondents had experienced a shortage of protamine, which is a common medicine used in cardiac surgery that has been affected by a national shortage since 2011.

**Table 2:** The top 10 drugs in shortage in 2016.

Rank	Drugs	Respondents
1	Protamine	129
2	Sodium dimercaptopropane sulfonate	92
3	Pralidoxime chloride	89
4	Sodium thiosulfate	82
5	Methylene blue	67
6	Flumazenil	50
7	Acetamide	46
8	Urokinase	42
9	Posterior pituitary	41
10	Propafenone	37

Although 28 respondents reported no shortages among the 25 emergency drugs, 6 respondents provided information on other drugs in shortage. In total, 32 respondents listed 7 extra emergency drugs (i.e., antivenin, atropine, penehyclidine, human tetanus immunoglobulin, tetanus antitoxin, oxytocin and vecuronium bromide). Among the additional 7 drugs, 5 were national essential medicines, and 4 had a daily cost less than 15 RMB.

### The characteristics of drugs in shortage

**Fig 3:**
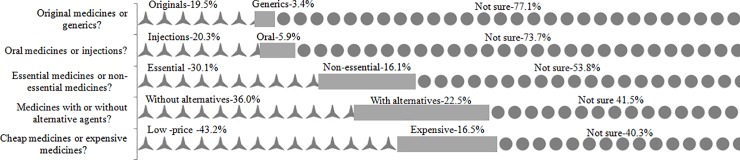
Ratios of respondents’ answers regarding the characteristics of the shortage drugs.

Most respondents agreed that original medicines, injections, essential medicines, medicines without alternative agents and cheap medicines were more susceptible to shortages than generics, orals, nonessential medicines, medicines with alternative agents and expensive medicines, respectively (Fig 3). However, more than half of the respondents chose the answer “not sure”, especially for the first three comparisons.

### The effects of drug shortages

**Fig 4:**
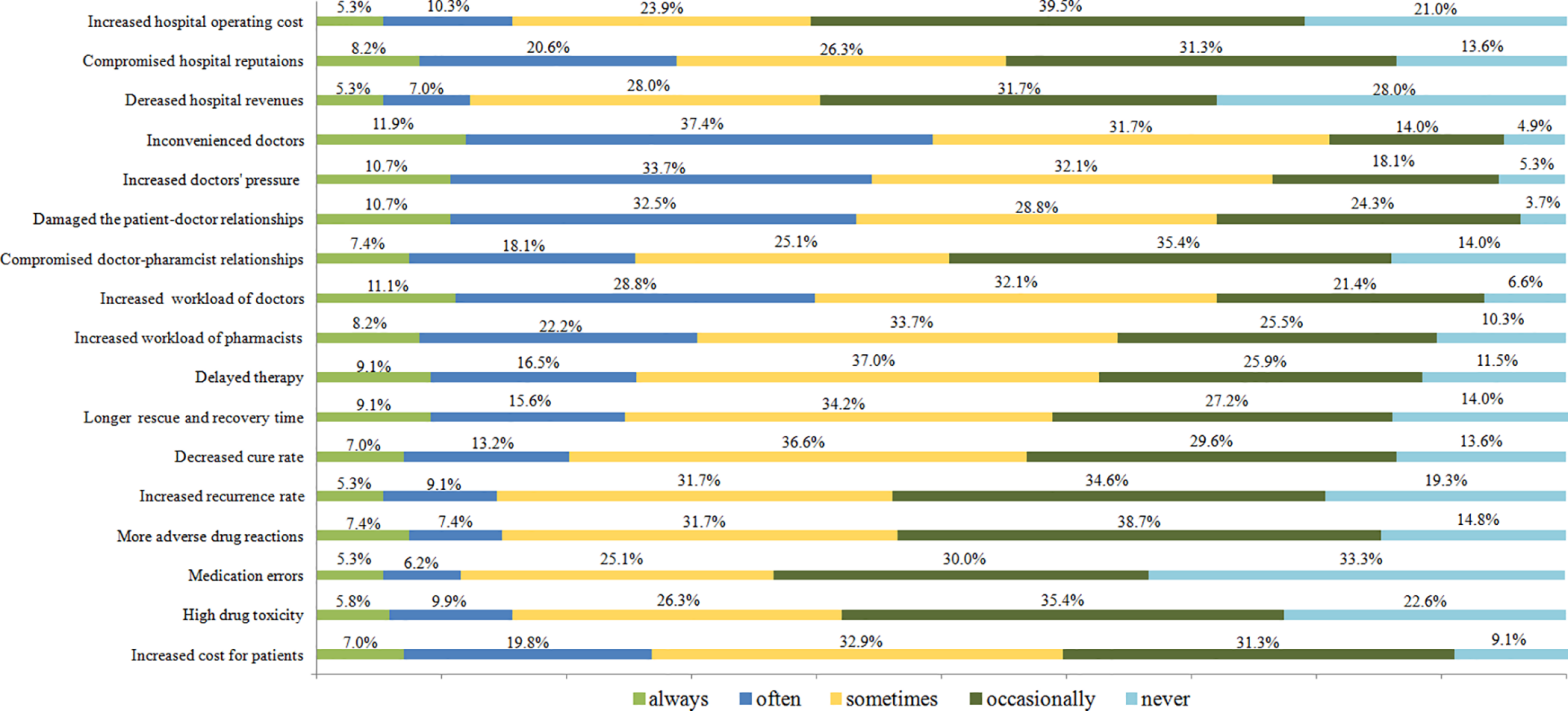
The impact of drug shortages.

The emergency physicians’ responses to the questions concerning the effects of drug shortages on hospitals, healthcare workers and patients are displayed in Fig 4.

At the hospital level, a compromised hospital reputation was indicated by 55.1% of the respondents as a consequence of drug shortages that occurred always, often, or sometimes. However, the majority of respondents thought drug shortages occasionally or never increased hospital operating costs or decreased hospital revenues.

At the medical staff level, the respondents indicated that drug shortages always, often, or sometimes caused inconvenience to physicians (81.1%), increased physician pressure (76.5%), and damaged patient-doctor relationships (72.0%).

At the patient level, the majority of the respondents indicated that drug shortages always, often, or sometimes delayed therapy (62.6%), led to longer rescue and recovery times (58.9%) and increased patient costs (58.7%). However, most respondents believed that shortage drugs never or occasionally led to an increased recurrence rate (53.9%), more adverse drug reactions due to use of alternative agents (53.5%), medication errors (63.3%) or high drug toxicity (58.0%).

### Sensitivity analysis

Based on the hospital information (hospital location, hospital level, hospital type and number of inpatient beds) provided by the respondents, we were able to determine that the 236 respondents were from at least 171 different hospitals. Upon analyzing the data from the 171 different hospitals, no indication of any differences between the results of the 171 samples and the total 236 samples was found (details provided in S2 Text). This analysis suggested that our primary findings were unlikely to be caused by sample clustering.

## Discussion

This study evaluated emergency drug shortages in China from the perspectives of physicians working in the emergency departments of Chinese hospitals. In total, 236 physicians from 29 of 31 provinces participated in the study. Their experiences with emergency drug shortages and the effects of these shortages on patients, health professionals and hospitals were characterized.

The situation of emergency drug shortages was characterized as substantial, and the number of shortage emergency drugs varied significantly across regions and hospitals. Hospitals in the eastern and western regions of China had more emergency drugs in shortage than those in the central region, even though the eastern and western regions are the most and least developed regions of China, respectively, especially regarding hospitals with a high number of inpatient beds (≥800). This finding may have occurred because the eastern region is the most populated area in China, which causes a greater demand for emergency drugs than the other regions. The severe drug shortage problem in the western region may be ascribed to its lower economy and poor traffic conditions. Similar results were reported by Yang et al [27], who performed a cross-sectional study of township hospitals in China and found that the availability of medicines in central China was better than that in the eastern and western regions. In addition, they noted that secondary hospitals experienced more severe shortages of emergency drugs than tertiary hospitals. Similar results were generated by Yang et al [21] and Chen et al [28]. Yang et al [21] argued that when the demand for certain drugs exceeded the supply in the whole market, wholesalers satisfied the requirements of tertiary hospitals before those of secondary hospitals because the tertiary hospitals had greater negotiating power than the secondary hospitals.

Emergency drug shortages in China mostly involve cheap national essential drugs without alternative agents. All 25 emergency drugs listed in the survey had a daily cost of less than 15 RMB, and 5 of the top 10 mentioned by physicians cost less than 5 RMB. The drug price is determined by the local government based on a centralized bidding and purchase policy. This price usually remains constant for a couple of years. However, over time, higher pharmaceutical production standards and increased raw material prices and labor wages lead to higher costs and lower profits for pharmaceutical companies [29]. Furthermore, shortages have occurred when pharmaceutical companies adjust the production line to reduce or stop producing low-profit drugs. This is similar to the reported cause of cheap drug shortages in Europe or the U.S. [30–33]. Medicines without alternative agents have more shortages than medicines with alternatives, perhaps because most emergency drugs (23/25) cannot be substituted or fully substituted. For example, metadoxine can be used as a substitute for naloxone hydrochloride for acute alcoholism only when patients do not have bronchial asthma. A shortage of drugs without alternative agents can have severe consequences for patients. For example, for patients with acute cerebral infarction who receive delayed treatment or are left untreated, more severe brain tissue necrosis occurs, and more brain function is eventually lost [34]. The shortage of essential medicines is another important issue in China. Twenty-two of the 25 and 9 of the top 10 drugs in shortage were essential medicines. As stated by the World Health Organization (WHO), essential medicines “ought to be available at all times, in the proper dosage forms, to all segments of society” [35]. The low availability of essential medicines may pose a serious threat to public health in China. Thus, the government needs to pay more attention to this problem in the future.

When drugs cannot be delivered to patients in emergency situations, every stakeholder of the entire medical system is affected [30]. The common clinical effect of emergency drug shortages on patients is delayed treatment. The top drug in shortage mentioned by respondents is protamine, which has been in short supply since 2011, and consequently, surgical treatments for many patients with heart disease have been delayed in China. More seriously, sometimes the condition of the patient deteriorates rapidly, and the life of the patient may be at risk in an emergency situation if treatment is delayed. For example, antivenom is a requisite medicine for the treatment of snakebite envenoming. Shortage of antivenin may cause permanent disability or death. Although alternative medicines can be used, their application may expose patients to a greater risk of a low cure rate. For instance, the alternative medicines for pralidoxime chloride are pralidoxime iodide and atropine, both of which result in poor treatment efficacy and a poor prognosis [36]. Medical staff, especially physicians, are affected by drug shortages. Physicians have to use complex suboptimal treatment and learn alternative treatments, such as hemodialysis for acute organophosphate poisoning when pralidoxime is unavailable. Increased pressure is another consideration because emergency physicians usually have little time to choose alternative therapies. Additionally, drug shortages affect hospitals. The shortages of emergency drugs vary in each hospital [28]. Some medicines may be in shortage in one hospital but available in other hospitals. If treatment is delayed or the patient’s condition deteriorates because of the shortage, patients or their families may blame the hospital and complain to others. This situation could cause a poor reputation for that hospital. However, the financial burden of hospitals in China does not appear to have increased much due to drug shortages, which has been an important problem for hospitals in the U.S. or European countries [35]. Possible reasons may be that hospitals in China do not take this shortage problem seriously and that these hospitals do not pay workers for the extra working hours associated with managing drug shortages [21]. In the U.S. and Europe, the labor costs for dealing with drug shortages are large. For example, the labor cost for dealing with drug shortages in U.S. hospitals was estimated to be $16 million annually [37].

This study had several limitations. Our results were limited by selection and recall bias. Although we had a low response rate, our respondents were from 29 of 31 provinces in mainland China, and thus, these results represent the drug shortage situation across the entire country. Our respondents were emergency physicians, and thus, they might be more familiar with the effects of drug shortages on themselves or on their patients than on hospitals and other healthcare workers. In addition, they may overestimate the effects of drug shortages on themselves. Moreover, because the results only capture the perceptions of the respondents, they may not provide a fully accurate picture of the effects of emergency drug shortages in China.

## Conclusions

This study measured the levels of emergency drug shortages in China. Overall, the shortage of emergency drugs was critical and varied between different regions and different hospital levels. Currently, emergency drug shortages in China mostly involve cheap medicines. The shortage problem continues to affect the entire healthcare system, causing delayed therapy, longer rescue and recovery times and increased economic burdens for patients, inconvenience for physicians, and compromised reputations for hospitals.

According to our findings, identifying the underlying causes of drug shortages and developing long-term intervention strategies are very important goals. More quantitative research is needed to validate these findings in the future.

## Supporting information

S1 Text Questionnaire for emergency drug shortages. (DOC)

S2 Text Sensitivity test. (DOCX)
