# Supplementary material for: The current status and effects of emergency drug shortages in China: Perceptions of emergency department physicians
Source: PLoS One. 2018 Oct 9;13(10):e0205238. doi: 10.1371/journal.pone.0205238 (PMC6177176; doi:10.1371/journal.pone.0205238)
Supplement: S1 Text — (DOC) [file pone.0205238.s001.doc]

## Supplement S1

**Explanatory Statement for the Participants**

Dear Mr./Mrs./Miss:

This project is launched by the Department of Pharmacy Administration and Clinical Pharmacy, School of Pharmacy, Xi’an Jiao tong University. It aimed to explore the current status and impacts of emergency drug shortages in China.

As you are physicians in the emergency department, you are most familiar with emergency drug shortages in your department. Your cooperation is critical to accomplish this project. While requesting your support for this study, we assure you that any information related to your identification will be kept strictly confidential and will not be disclosed at any stage of the study. All the information you provide will be used only for research. Participation in this project is entirely voluntary.

To become part of this study as a respondent, you agreed that

1. You have read the information given above;

2. You voluntarily agree to be part of this research and provide necessary information.

A copy of the final research outcomes will be provided on request. If you have any queries regarding this research, please feel free to contact us.

We highly appreciate your cooperation in supporting this research. Thank you!

The Department of Pharmacy Administration and Clinical Pharmacy

School of Pharmacy, Xi’an Jiao tong University

Xi’an, Shaanxi Province, China

Phone: 029-82655132

Email: [yangcj@xjtu.edu.cn](mailto:yangcj@xjtu.edu.cn)

**Questionnaire for Emergency Drug Shortages**

**Part I Demographics：**

1. Hospital location:

Provinces: Cities: Districts/Counties:

2. Hospital level:

(1) Primary hospital (2) Secondary hospital (3) Tertiary hospitals

3. Hospital type

(1) General hospital (2) Specialized Hospital (3) Traditional Chinese medicine hospital

(4) Minority hospital (5) TCM-WM hospital

4. Number of inpatient beds

(1) < 100 (2) 100 to 199 (3) 200 to 499 (4) 500 to 799 (5) 800 or more

**Part II Current situation of drug shortage**

5. How often does drug shortage happen in your department on average？

(1) Every day (2) Every week (3) Every month (4) A longer period of time (longer than one month) (5) Never appears

6. Choose the trade names of drugs in shortage during last one year in your department.

| Protamine | Glucose | Heparin | Methylene blue |
| --- | --- | --- | --- |
| Acetamide | Urokinase | Metoprolol | Aminocaproic acid |
| Propafenone | Flumazenil | Metaraminol | Posterior pituitary |
| Monarkite | Mannitol | Norepinephrine | Sodium nitroprusside |
| Magnesium | Dopamine | Phentolamine | Sodium thiosulfate |
| Potassium chloride | Naloxone hydrochloride | Sodium dimercaptopropane sulfonate | Sodium bicarbonate |
| Pralidoxime chloride | Others If other, please specify） | | |

7. Which kinds of medicines suffer more shortages?

(1) Original medicines or generics?

□Original medicines □Generics □Not sure

(2) Oral medicines or injections?

□Oral medicines □Injections □Not sure

(3) Essential medicines or non-essential medicines?

□Essential medicines □Non-essential medicines □Not sure

(4) Medicines with or without alternative agents?

□Medicines with alternative agents □Medicines without alternative agents □Not sure

(5) Cheap medicines or expensive medicines?

□Cheap medicines □Expensive medicines □Not sure

**Part III The impact**

8. What impact has a drug shortage caused in your department?

| Impact on hospital | never | occasionally | | sometimes | often | always |
| --- | --- | --- | --- | --- | --- | --- |
| (1) Increased operating costs of hospital |  |  | |  |  |  |
| (2) Compromised hospital reputations |  |  | |  |  |  |
| (3) Decreased hospital revenues |  |  | |  |  |  |
| Impact on doctor and pharmacist | | | | | | |
| 1. Inconvenienced doctors |  |  | |  |  |  |
| 1. Increased doctors’ pressure |  |  | |  |  |  |
| 1. Damaged the patient-doctor relationships |  |  | |  |  |  |
| 1. Compromised doctor-pharmacist relationships |  |  | |  |  |  |
| 1. Increased workload of doctors |  |  | |  |  |  |
| (6) Increased workload of pharmacists |  |  | |  |  |  |
| Impact on patient | | | | | | |
| 1. Delayed therapy |  | |  |  |  |  |
| 1. Longer rescue and recovery time |  | |  |  |  |  |
| 1. Decreased cure rate |  | |  |  |  |  |
| 1. Increased the recurrence rate |  | |  |  |  |  |
| 1. More adverse drug reactions caused by using alternative drugs |  | |  |  |  |  |
| 1. Medication error |  | |  |  |  |  |
| 1. High drug toxicity |  | |  |  |  |  |
| 1. Increased cost of patients |  | |  |  |  |  |

**Thanks for your cooperation!**
