# Supplementary material for: The current status and effects of emergency drug shortages in China: Perceptions of emergency department physicians
Source: PLoS One. 2018 Oct 9;13(10):e0205238. doi: 10.1371/journal.pone.0205238 (PMC6177176; doi:10.1371/journal.pone.0205238)
Supplement: S2 Text — (DOCX) [file pone.0205238.s002.docx]

**Sensitivity test**

Based on the hospital information provided by the respondents, we were able to determine that the 236 respondents were from at least 171 different hospitals. For respondents provided the same information of hospitals may come from different hospitals, or also may came from the same hospital. To find out the maximal cluster effects, we assumed that the participants who shared the same hospital information came from the same hospital. Then we compared the results of two different scenarios: 1) Scenario A: including all participants in the analysis (as done in the text) provided 236 samples. 2) Scenario B: If several participants shared the same hospital information, we randomly choose one participant’s result for the analysis, which obtained 171 samples.

The following results were obtained for scenarios A and B.

1. **Characteristics of the hospitals where the respondents worked**

As shown in Table 1, slight variation was found between scenarios A and B.

Table 1. Characteristics of the hospitals where the respondents worked

| **Characteristic** | **Scenario A** | **Scenario B** |
| --- | --- | --- |
|  | **Respondents (*n*=236)** | **Respondents (*n*=171)** |
| Hospital location (n, %) |  |  |
| Eastern China | 73 (30.9) | 58 (33.9) |
| Central China | 67 (28.4) | 44 (25.7) |
| Western China | 96 (40.7) | 69 (40.4) |
| Hospital level (n, %) |  |  |
| Secondary hospitals | 52 (22.0) | 44 (25.7) |
| Tertiary hospitals | 184 (78.0) | 127 (74.3) |
| Hospital type (n, %) |  |  |
| General | 203 (86.0) | 143 (83.6) |
| Specialized | 9 (3.8) | 9 (5.3%) |
| Traditional Chinese Medicine | 18 (7.6) | 13 (7.6) |
| Minority | 1 (0.4) | 1 (0.6) |
| TCM-WM | 5 (2.2) | 5 (2.9) |
| Number of inpatient beds (n, %) |  |  |
| <100 | 3 (1.3) | 3 (1.8) |
| 100-199 | 11 (4.7) | 9 (5.3) |
| 200-499 | 21 (8.9) | 20 (11.7) |
| 500-799 | 47 (19.9) | 39 (22.8) |
| ≥800 | 154 (65.2) | 100 (58.5) |

1. **The status of drug shortages**

Among the respondents, in both scenarios, more than 90% of emergency physicians had experienced drug shortages during the last year (Table 2). In response to the question “how often does a drug shortage occur in your department on average”, 65.7% (72%) of the respondents reported that drug shortages occurred every month or more frequently in scenario A (B).

In both scenarios, no significant differences in the frequency of drug shortages existed between the different regions or hospital levels according to the Chi-square test.

Table 2. The frequency of drug shortages reported by respondents

| **Frequency** | **Respondents (*n*, %)** | |
| --- | --- | --- |
|  | **Scenario A** | **Scenario B** |
| Every day | 19 (8.1) | 17 (9.9) |
| Every week | 20 (21.2) | 40 (23.4) |
| Every month | 86 (36.4) | 66 (38.6) |
| Longer than one month | 59 (25.0) | 38 (22.2) |
| Never appears | 22 (9.3) | 10 (5.8) |

Table 3. The number of shortage drugs reported by respondents

| **The number of shortage drugs** | **Respondents (*n*, %)** | |
| --- | --- | --- |
|  | **Scenario A** | **Scenario B** |
| ≥10 | 16 (6.8) | 12 (7.0) |
| 5-9 | 75 (31.8) | 61 (35.7) |
| 1-4 | 117 (49.5) | 90 (52.6) |
| 0 | 28 (11.9) | 8 (4.7) |

Among the 25 listed medicines, the top 10 drugs in shortage reported by the respondents were the same in both scenarios (Table 4). Additionally, 7 out of 10 drugs had the same rank in scenarios A and B. In both scenarios, more than 50% of the participants reported protamine.

Table 4. The top 10 drugs that were in shortage in 2016 reported by the respondents

| **Rank** | **Drugs ( *n*, % )** | |
| --- | --- | --- |
|  | **Scenario A** | **Scenario B** |
| 1 | Protamine (129, 54.7) | Protamine (96, 56.1) |
| 2 | Sodium dimercaptopropane sulfonate (92, 39.0) | Sodium dimercaptopropane sulfonate (71, 41.5) |
| 3 | Pralidoxime chloride (89, 37.7) | Pralidoxime chloride (71, 41.5) |
| 4 | Sodium thiosulfate (82, 34.7) | Sodium thiosulfate (61, 35.7) |
| 5 | Methylene blue (67, 28.4) | Methylene blue (51, 29.8) |
| 6 | Flumazenil (50, 21.2) | Flumazenil (41, 24.0) |
| 7 | *Acetamide (46, 19.5)* | *Urokinase (41, 24.0)* |
| 8 | *Urokinase (42, 17.8)* | *Propafenone (32, 18.7)* |
| 9 | Posterior pituitary (41, 17.4) | Posterior pituitary (32, 18.7) |
| 10 | *Propafenone (37, 15.7)* | *Acetamide (30, 17.5)* |

1. **Characteristics of the drug shortages**

In both scenarios, most respondents agreed that original medicines, injections, essential medicines, medicines without alternative agents and cheap medicines were more susceptible to shortages than generics, orals, non-essential medicines, medicines with alternative agents and expensive medicines, respectively (Figures 1 and 2).


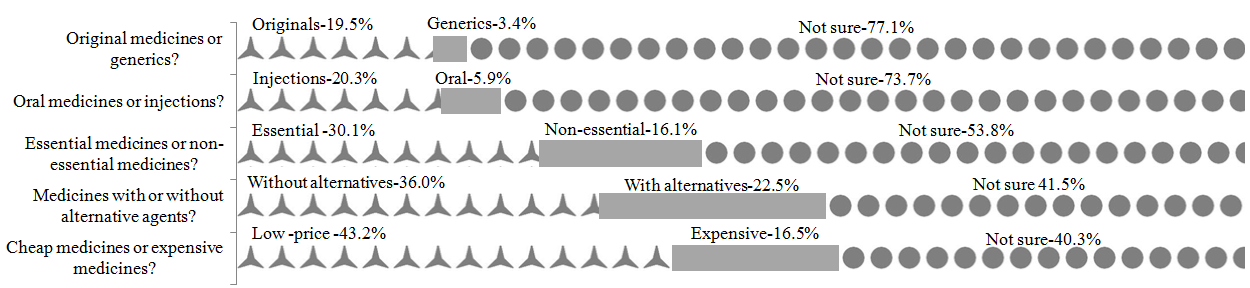


Figure 1. Ratio of respondents’ choices of the characteristics of shortage drugs in Scenario A


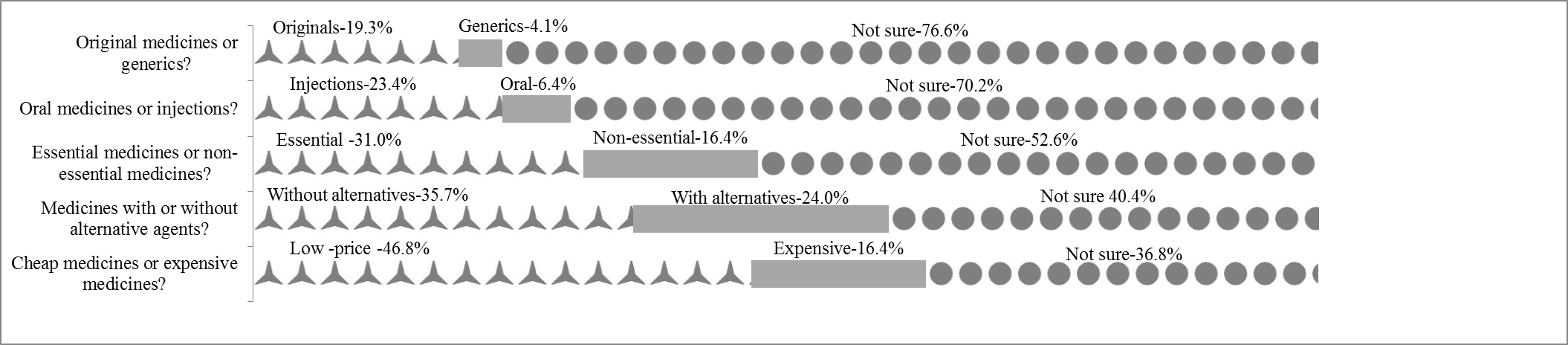
Figure 2. Ratio of respondents’ choices of the characteristics of shortage drugs in Scenario B

1. **The effect of drug shortages**

From Figure 3 and 4, we found that most of the effects caused by drug shortages were similar in both scenarios A and B. The most common effect on hospitals due to drug shortages is a compromised hospital reputation, which was reported by 55.1% (59.1%) of the respondents in scenario A (B). The top three effects on medical staff were inconvenience to physicians, increased physician pressure, and damage of patient-doctor relationships, which were reported by 81.1% (84.8%), 76.5% (81.2%) and 72.0% (77.1%) of the respondents in scenario A (B), respectively. The common clinical impacts of emergency drug shortages on patients were delaying treatment, causing longer rescue times and increasing patient costs, which were reported by 62.6% (64.6%), 58.9% (58.9) and 58.7% (59.7%) of the respondents in scenario A (B), respectively.


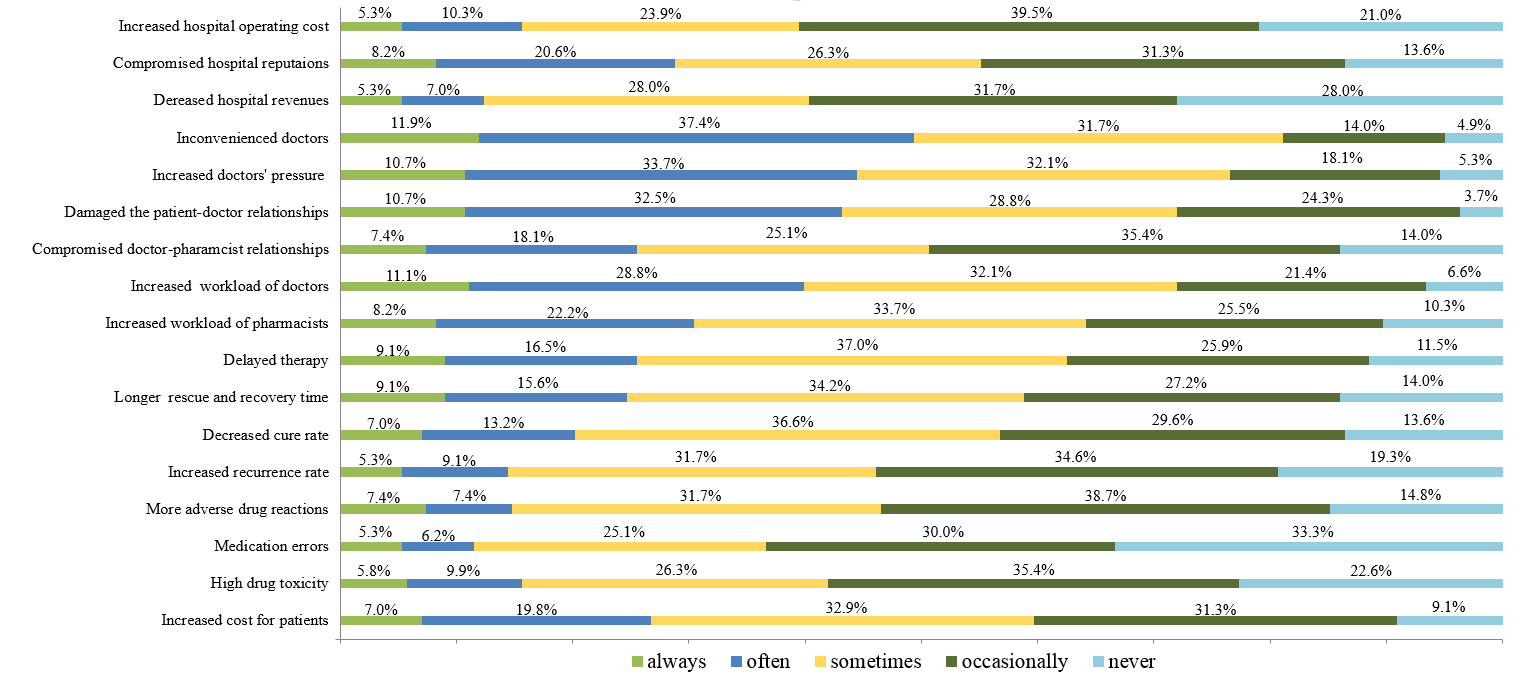


Figure 3. The impact of drug shortages in scenario A


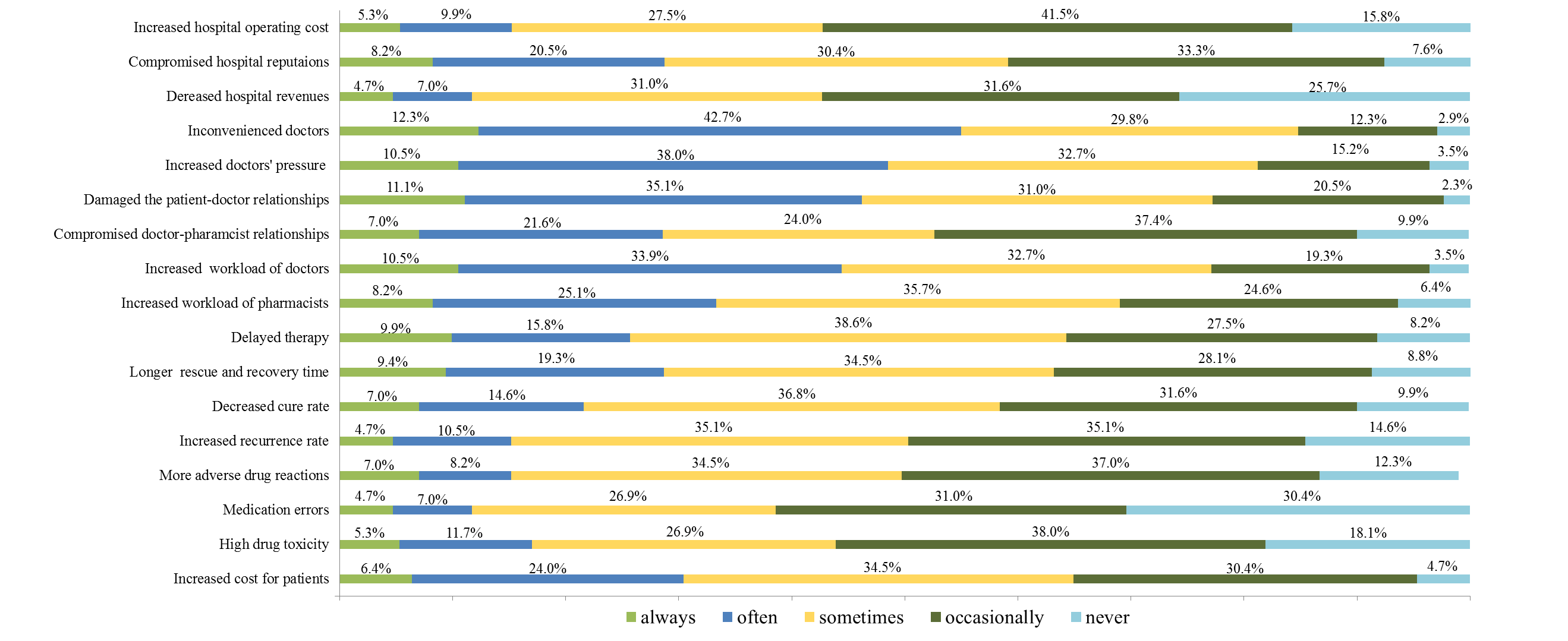


Figure 4. The impact of drug shortages in scenario B

Based on the above results obtained for scenarios A and B, we did not observe significant differences. Therefore, we believed that the sample cluster phenomenon was not impactful and would not affect the results.
